# Preliminary study of standardized semiquantitative method for ultrasonographic breast composition assessment

**DOI:** 10.1007/s10396-024-01463-7

**Published:** 2024-05-03

**Authors:** Takayoshi Uematsu, Kazuaki Nakashima, Hatsuko Nasu, Tatsuya Igarashi, Yukiko Okayama, Akifumi Notsu

**Affiliations:** 1 Department of Breast Imaging and Breast Intervention Radiology, Shizuoka Cancer Center Hospital, 1007 Shimonagakubo, Nagaizumi, Shizuoka, 411-8777 Japan; 2 Department of Radiology, Hamamatsu University School of Medicine, Shizuoka, Japan; 3 Department of Radiology, Fujieda Municipal General Hospital, Shizuoka, Japan; 4 Department of Clinical Physiology, Shizuoka Cancer Center Hospital, Shizuoka, Japan; 5 Clinical Research Center, Shizuoka Cancer Center Hospital, Shizuoka, Japan

**Keywords:** Breast composition, Ultrasonography, Mammography, Glandular tissue component, Semiquantitative analysis

## Abstract

**Purpose:**

To develop a classification tree via semiquantitative analysis for ultrasonographic breast composition assessment using routine breast ultrasonography examination images.

**Methods:**

This study retrospectively enrolled 100 consecutive normal women who underwent screening mammography and supplemental ultrasonography. Based on sonographic breast composition, the patients’ breasts were classified as nondense or dense, which were correlated with mammographic breast composition. Ultrasonographic breast composition was classified based on the fibroglandular tissue (FGT) thickness-to-subcutaneous fat and retromammary fat (FAT) thickness ratio. In addition, the presence of a high glandular tissue component (GTC) in FGT or the presence of evident fat lobules in FGT was investigated. The cutoff point between the nondense and dense breasts was calculated from the area under the curve (AUC).

**Results:**

All cases with a high GTC were dense breasts, and all cases with evident fat lobules in the FGT were nondense breasts. The AUC of the FGT thickness-to-FAT ratio of all cases, the group without a high GTC, the group without evident fat lobules in the FGT, and the group without a high GTC or evident fat lobules in the FGT were 0.93, 0.94, 0.99, and 1, respectively.

**Conclusion:**

The presence of a high GTC indicated dense breasts, and the presence of evident fat lobules in the FGT represented nondense breasts. For the remaining cases, the cutoff point of the FGT thickness-to-FAT thickness ratio was 0.93 for ultrasonographic two-grade scale breast composition assessment with 100% accuracy.

## Introduction

Breast cancer mortality in Japan has not decreased despite the implementation of population-based screening mammography for > 20 years. Screening mammography may not be suitable for Japanese women who often have dense breasts, thereby decreasing mammography sensitivity because of masking [1]. The Japan Strategic Anticancer Randomized Trial (J-START) is the first large-scale, randomized controlled trial of supplemental breast screening ultrasonography for women aged 40–49 years. It evaluated the efficacy of screening mammography with supplemental ultrasonography. Results showed that supplemental ultrasonography not only increased the sensitivity and detection rate of early invasive cancers but also reduced the development of interval cancers in the intervention group compared with the control group [2, 3]. To date, J-START has not been fully evaluated, and further observations of the effect of supplemental ultrasonography screening on mortality are ongoing. Mortality rates are the most important parameters for assessing the effectiveness of supplemental breast ultrasonography screening. However, the preliminary results of J-START are important in guiding women with dense breasts in selecting individualized breast cancer screening methods. Based on the J-START results, a next-generation approach to breast cancer screening in Japan can introduce supplemental screening ultrasonography [1, 4, 5]. In addition, 19% of Japanese women have already undergone breast ultrasonography screening in 2019 [6]. Further, 40% of local governments have performed breast ultrasonography for population-based breast cancer screening in 2020 [7]. Thus, breast ultrasonography is a common breast cancer screening modality in Japan. Therefore, some Japanese women with nondense breasts can undergo ultrasonography-based breast cancer screening based on their preferences. However, the number of certified sonographers and physicians in Japan is limited [1]. Thus, supplemental ultrasonography-based breast cancer screening might be currently limited to women with dense breast tissues in Japan. Nevertheless, patient preferences must be respected.

To overcome this issue, patients must be informed that screening mammography is significantly more effective for women with nondense breasts, particularly those with fatty breasts, and that ultrasonography should be performed as a supplemental screening modality in women with dense breasts. However, women who undergo ultrasonography-based breast cancer screening, but not screening mammography, have no information about whether their breasts are dense. Therefore, a standardized method for ultrasonographic breast composition assessment in routine breast ultrasonography examination images is required.

To the best of our knowledge, no studies have quantified ultrasonographic breast composition assessment using routine breast ultrasonography examination images and compared it with mammographic breast composition. The current study aimed to develop a classification tree via semiquantitative analysis for ultrasonographic breast composition assessment (dense or nondense) using routine breast ultrasonography examination images based on mammographic breast composition assessment.

## Materials and methods

We retrospectively collected data from 100 consecutive normal female Japanese patients who underwent screening mammography and supplemental screening breast ultrasonography on the same day between January 2021 and June 2021 at Shizuoka Cancer Center Hospital. The participants’ median age was 57 (range: 41–69) years, and 75% (n = 75/100) were postmenopausal. The institutional review board of our institution approved this retrospective study, and the need for informed consent was waived.

### Ultrasonography scanning and assessment

**Fig. 1:**
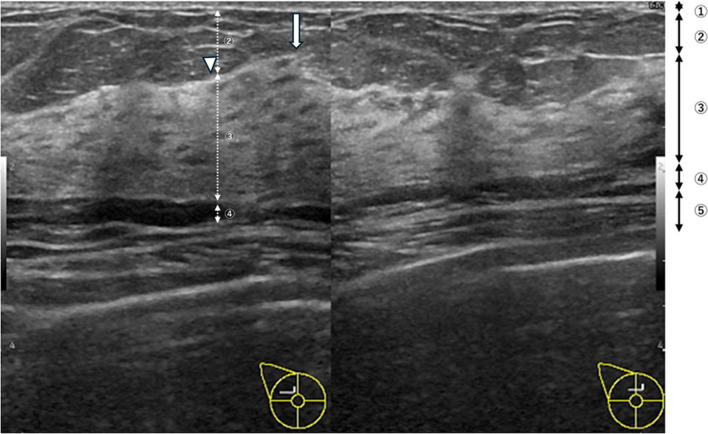
Routine breast ultrasonography examination image of the right breast. The numbers on the image correspond to the numbered anatomical structures: 1, skin; 2, subcutaneous fat; 3, fibroglandular tissue (FGT); 4, retromammary fat; and 5, chest muscle. This image does not show any site with flat anterior superficial fascia including the thickest FGT layer. The better representative point (arrowhead) is halfway between a site with a concave anterior superficial fascia and a site with a convex anterior superficial fascia due to the Cooper ligament. FGT thickness-to-FAT thickness ratio = FGT thickness (3)/FAT thickness (2 + 4). FAT means subcutaneous fat (2) and retromammary fat (4). The arrow indicates a site with a convex anterior superficial fascia due to the Cooper ligament that is not suitable for the representative point

All ultrasonography examinations were performed in accordance with the ISO 15189 standard by one of 10 certified sonographers with 1–19 years of experience in breast ultrasonography using the ARIETTA 850, ARIETTA 70, or Prosound F75 system (Hitachi, Ltd., Tokyo, Japan); Aplio XG SSA-790A or 500 system (Toshiba Medical Systems, Tokyo, Japan); or LOGIQE-10 (GE HealthCare, Tokyo, Japan) with linear transducers (10–18 MHz). The screening breast ultrasonography technique was standardized. The sonographers were instructed to begin by scanning the left breast and then the right breast using the transverse orientations. In a routine screening examination, the sonographers were required to take representative images that included the thickest layer of the fibroglandular tissue (FGT) in each breast (Fig. 1). Thus, radiologists could assess the distribution and amount of FGT in each breast after ultrasonography examinations. In addition, the representative images clearly showed five main layers including the skin, subcutaneous fat, FGT, retromammary fat, and chest muscle in the normal breast sonographic anatomy (Fig. 1). We referred to total fat (subcutaneous fat and retromammary fat) as FAT in the representative images.

In general, breast composition is defined according to FGT and FAT balance on breast imaging. Therefore, one breast radiologist evaluated ultrasonographic breast composition in each case, which was calculated as the FGT thickness-to-FAT thickness ratio, as follows:

FGT thickness-to-FAT thickness ratio = FGT thickness/FAT thickness (Fig. 1)

In order to reduce the measurement variability for calculating the FGT thickness-to-FAT thickness ratio, we determined that the best representative point would be a site with flat anterior superficial fascia, including the thickest layer of FGT. A site with convex anterior superficial fascia that is caused by a Cooper ligament would not be suitable as a representative point. In the absence of a site with flat anterior superficial fascia including the thickest layer of FGT, the better representative point would be a halfway site including the thickest layer of FGT between a site with concave anterior superficial fascia and a site with convex anterior superficial fascia.

**Fig. 2:**
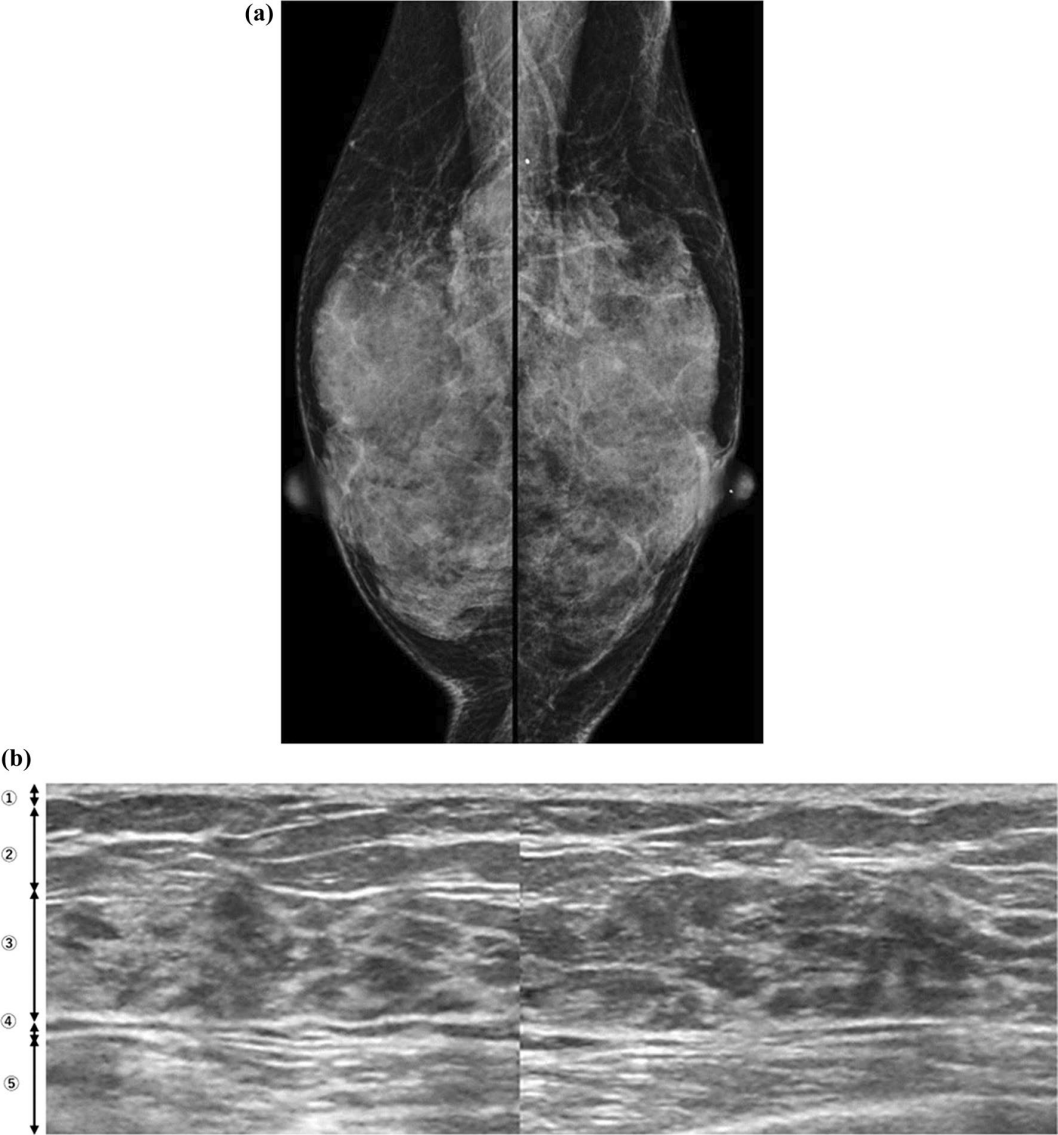
Mammographic dense breasts that are also ultrasonographic dense breasts with a high glandular tissue component in the fibroglandular tissue. **a** Mammograms in bilateral mediolateral oblique view showing extremely dense breasts. **b** This corresponding ultrasonography image shows a high glandular tissue component (GTC). Normal fibroglandular tissue (FGT) includes isoechoic dendritic structures that are equivalent to the GTC. 1, skin; 2, subcutaneous fat; 3, FGT; 4, retromammary fat; and 5, chest muscle

**Fig. 3:**
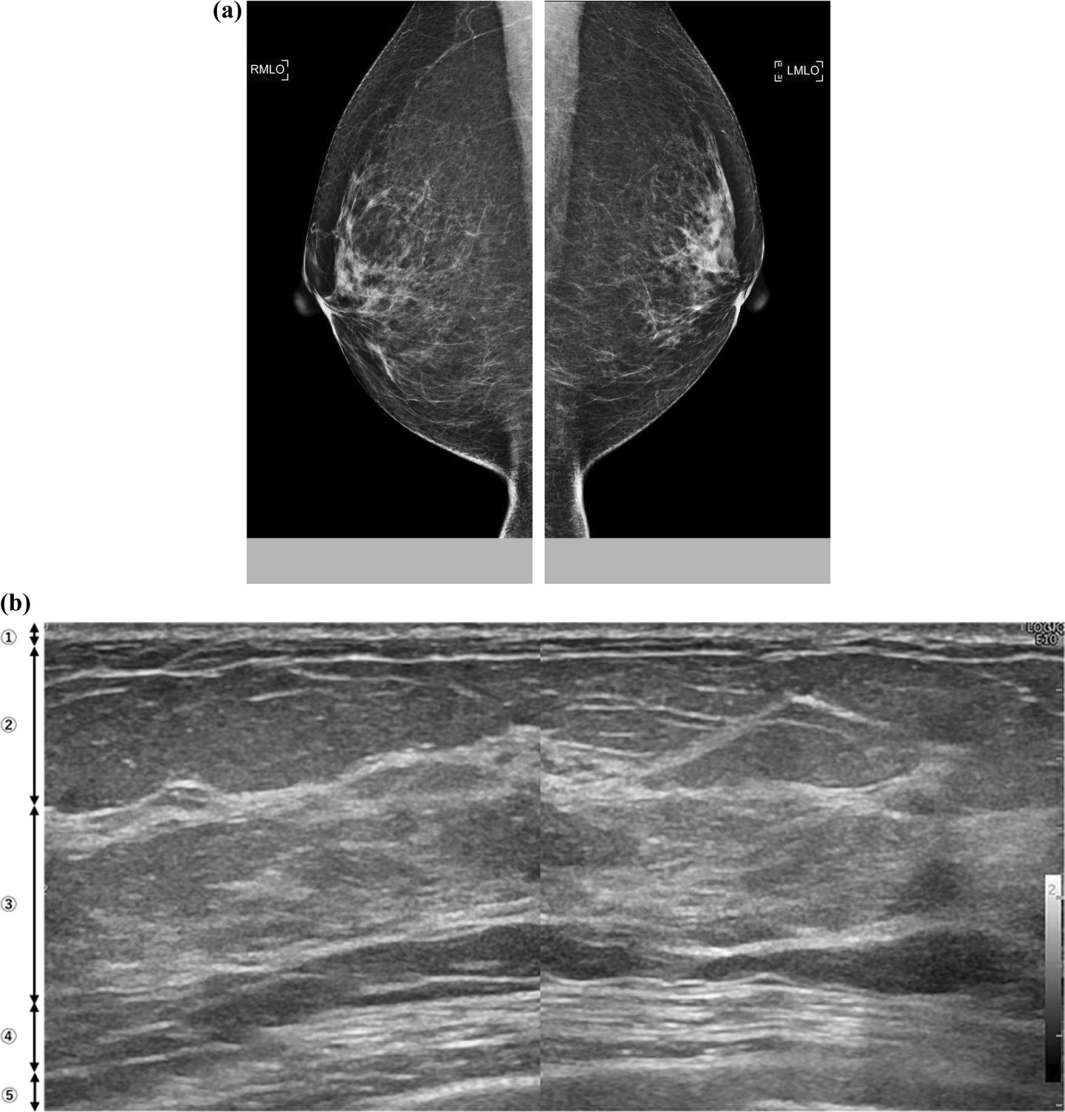
Mammographic nondense breasts that are also ultrasonographic nondense breasts with evident fat lobules in the fibroglandular tissue. **a** Mammograms in bilateral mediolateral oblique view showing scattered areas of fibroglandular density as nondense breasts. **b** This corresponding ultrasonography image shows evident fat lobules in the fibroglandular tissue (FGT). Normal FGT almost replaces fat lobules and has no GTC. 1, skin; 2, subcutaneous fat; 3, FGT; 4, retromammary fat; 5, chest muscle

In addition, the presence of a high glandular tissue component (GTC) in FGT was investigated because women with a higher mammographic breast composition have a greater GTC [8, 9] (Fig. 2). GTC can be dichotomized as low or high according to a GTC that represents 50% of the FGT. Fat lobules in the FGT, which are distinct from glandular tissues, were not counted in the GTC assessment [8]. Moreover, the presence of evident fat lobules in FGT, which are different from GTC, was examined because lobular involution usually occurs and FGT replaces fat with age (Fig. 3). Previous research has shown a positive association between nondense breasts, reflecting fat tissue, and complete lobular involution [9].

### Mammography screening and assessment

Mammograms with two standard imaging planes (mediolateral oblique and craniocaudal views) were obtained using a commercially available full-field digital mammography unit (Selenia Dimensions; Hologic, Bedford, MA). Three radiologists retrospectively reviewed all mammograms for mammographic breast composition assessment according to the new Japanese breast density classification system [10], which is a modification of the Breast Imaging-Reporting and Data System (BI-RADS) with a two-grade scale (nondense and dense breasts).

### Statistical analysis

A woman had a higher breast density if the density of one breast was different from that of the other. Thus, our analysis was performed at the examination level. All breasts with sonographic breast composition were classified as nondense or dense, and breast density was correlated with mammographic breast composition. Fisher’s exact test was used to compare the presence of a high GTC and evident fat lobules in FGT among mammographic breast composition groups. The receiver operating characteristic (ROC) curves of the FGT thickness-to-FAT thickness ratio was calculated, and the AUC was determined using the R statistical package version 4.3.2 (R Core Team, October 2023; www.r-project.org). The cutoff point between nondense and dense breasts was calculated using Youden’s index (highest value for [sensitivity + specificity − 1]).

## Results

Table 1 shows the association between the presence of a high GTC and evident fat lobules in the FGT and mammographic breast composition groups. The presence of a high GTC always indicated a dense breast, and the presence of evident fat lobules in the FGT always represented nondense breasts.

**Table 1:** Association between the presence of a high GTC and evident fat lobules in the FGT and mammographic breast composition groups

Variables	Mammographic breast composition		p value
	Nondense	Dense	
GTC in FGT			< .0001
Low	46	16	
High	0	38	
Evident fat lobules in the FGT			< .0001
Negative	25	54	
Positive	21	0	

*GTC* glandular tissue component, *FGT* fibroglandular tissue

**Fig. 4:**
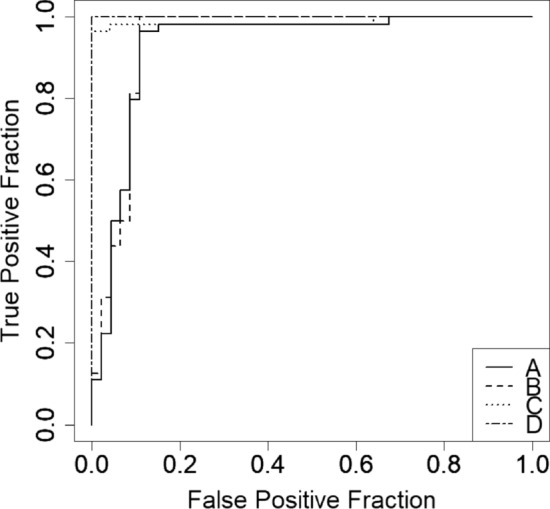
Receiver operating characteristic curves of the fibroglandular tissue (FGT) thickness-to-FAT thickness ratio of all cases (A), the FGT thickness-to-FAT thickness ratio of the group without a high GTC (B), the FGT thickness-to-FAT thickness ratio of the group without massive fat lobules in the FGT (C), and the FGT thickness-to-FAT thickness ratio of the group without a high GTC or evident fat lobules in the FGT (D). The area under the curve of the FGT thickness-to-FAT thickness ratio of the group without a high GTC or evident fat lobules in the FGT (D) is 1, which is higher than that for the FGT thickness-to-FAT thickness ratio of all patients (A) (0.93), the group without a high glandular tissue component (B) (0.94), and the group without evident fat lobules in the FGT (C) (0.99). The overall diagnostic performance of all four classification methods is high

Figure 4 presents the ROC curves of the FGT thickness-to-FAT thickness ratio of all patients, the group without a high GTC, the group without evident fat lobules in the FGT, and the group without a high GTC or evident fat lobules in the FGT. All four curves showed nearly the same performance. The AUC of the FGT thickness-to-FAT thickness ratio of all patients, the group without a high GTC, the group without evident fat lobules in the FGT, and the group without a high GTC or evident fat lobules in the FGT was 0.93, 0.94, 0.99, and 1, respectively. The overall diagnostic performance for each of the four classification methods was high.

**Fig. 5:**
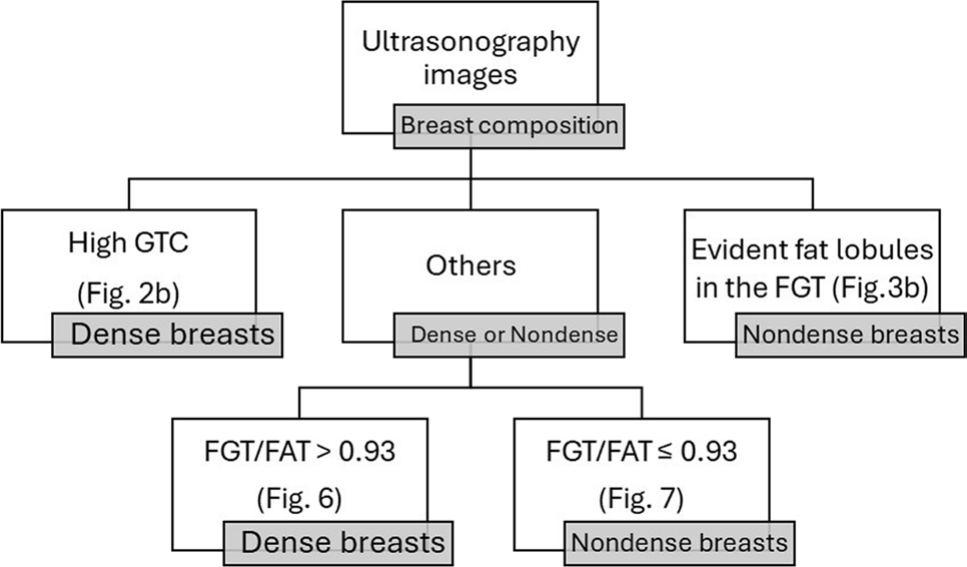
Classification tree for dense versus nondense breasts for routine breast ultrasonography examination images

**Fig. 6:**
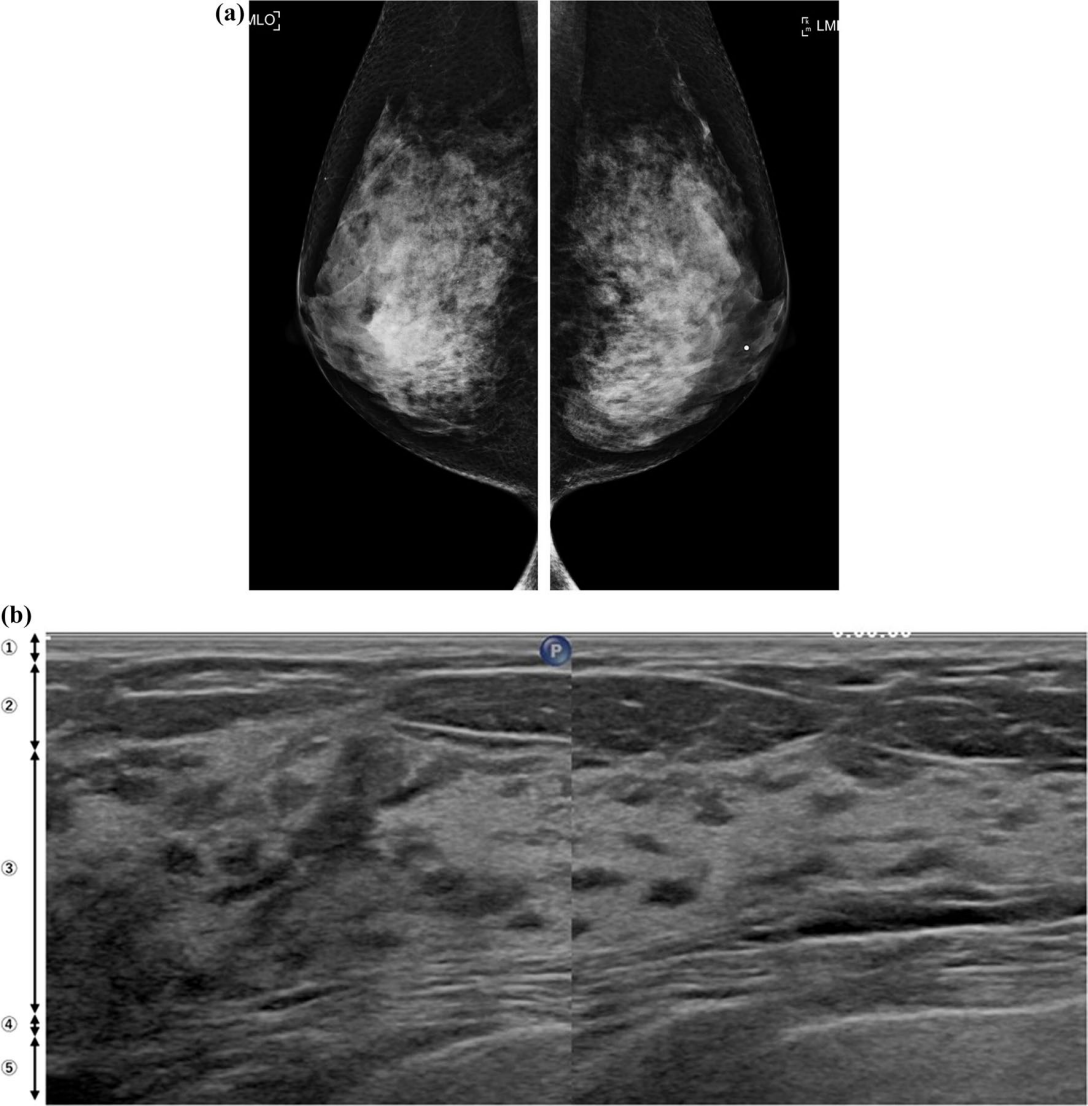
Mammographic dense breasts that are also ultrasonographic dense breasts without a high GTC but with a FGT thickness-to-FAT thickness ratio > 0.93. **a** Mammograms in bilateral mediolateral oblique view showing extremely dense breasts. **b** This corresponding ultrasonography image shows a FGT thickness-to-FAT thickness ratio > 0.93 without a high GTC. 1, skin; 2, subcutaneous fat; 3, FGT; 4, retromammary fat; 5, chest muscle

**Fig. 7:**
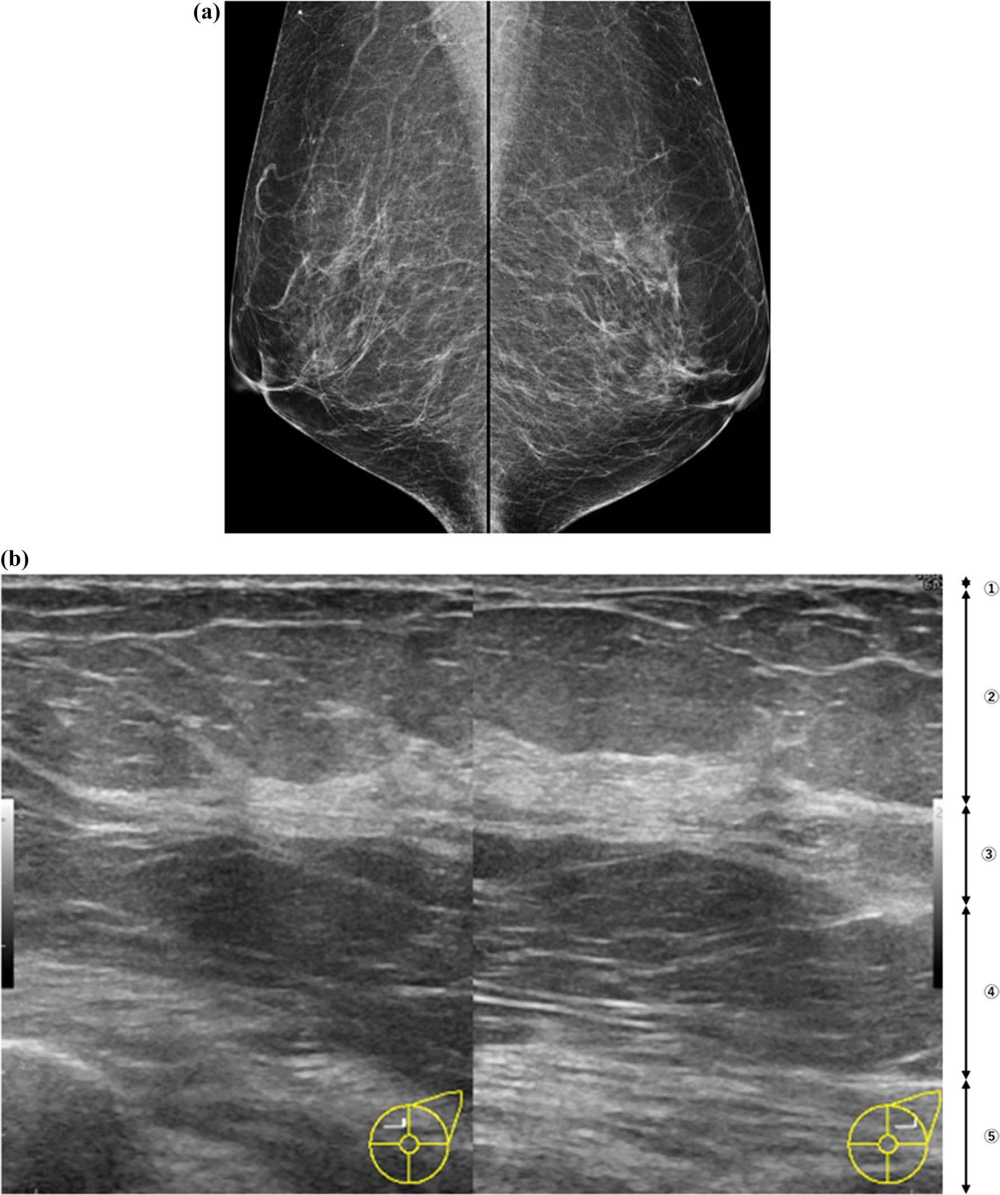
Mammographic nondense breasts that are also ultrasonographic nondense breasts without evident fat lobules in the fibroglandular tissue but with a FGT thickness-to-FAT thickness ratio ≤ 0.93. **a** Mammograms in bilateral mediolateral oblique view showing scattered areas of fibroglandular density as nondense breasts. **b** This corresponding ultrasonography image shows a FGT thickness-to-FAT thickness ratio ≤ 0.93 without evident fat lobules in the FGT. 1, skin; 2, subcutaneous fat; 3, FGT; 4, retromammary fat; 5, chest muscle

The cutoff point of the FGT thickness-to-FAT thickness ratio calculated using Youden’s index in all cases was 0.95, with a sensitivity of 89% and a specificity of 99%. The cutoff points were 0.96 for the group without a high GTC (sensitivity: 100%, specificity: 89%), 0.92 for the FGT thickness-to-FAT thickness ratio of the group without evident fat lobules in the FGT (sensitivity: 96%, specificity: 100%), and 0.93 for the group without a high GTC or evident fat lobules in the FGT (sensitivity: 100%, specificity: 100%). Thus, the classification tree (Fig. 5) comprising three criteria (a 0.93 cutoff point of the FGT thickness-to-FAT thickness ratio in the groups without a high GTC or evident fat lobules in the FGT) reached 100% accuracy (see Figs. 6, 7).

## Discussion

This study showed a simple and robust classification tree for differentiating dense from nondense breasts and validating a standardized semiquantitative method for ultrasonographic breast composition assessment in routine breast ultrasonography examination images. The presence of a high GTC always indicated mammographic dense breasts. The presence of evident fat lobules in the FGT always represented mammographic nondense breasts. The FGT thickness-to-FAT thickness ratio of the group without a high GTC or evident fat lobules in the FGT had the highest AUC at 1.00. The FGT thickness-to-FAT thickness ratio could be selected as a standardized semiquantitative method for ultrasonographic breast composition assessment. The cutoff point was 0.93 with 100% sensitivity and 100% specificity. The simple and robust classification tree with three criteria (presence of a high GTC, presence of evident fat lobules in the FGT, and a 0.93 cutoff point for the FGT thickness-to-FAT thickness ratio of the groups without a high GTC or evident fat lobules in the FGT) had a high accuracy of 100% for differentiating dense from nondense breasts on routine breast ultrasonography examination images.

A previous study using a real-time handheld ultrasound device showed a significant correlation between mammographic breast composition and ultrasonographic breast composition according to the BI-RADS terms [11]. However, the ultrasonographic breast composition according to the BI-RADS is classified as homogeneous background echotexture (fat), homogeneous background echotexture (fibroglandular), and heterogeneous background echotexture. These three categories correspond loosely to the four mammographic breast compositions [12]. Thus, four mammographic and three sonographic breast compositions do not have a one-to-one correspondence. Further, the ultrasonographic breast composition cannot be directly compared with the corresponding mammographic breast composition. Therefore, it might cause confusion and variability. In addition, the authors measured the ultrasonographic breast composition subjectively. Compared with that study, this study first focused on semiquantifying ultrasonographic breast composition by calculating the FGT thickness-to-FAT thickness ratio on routine breast ultrasonography examination images. In this study, the radiologist could classify the ultrasonographic breast composition based on a single image of the whole volume using a representative image that includes the thickest FGT layer in each breast (Fig. 1). Our method had higher reproducibility than the real-time handheld ultrasound method because of the separation of image acquisition and interpretation processes.

The association between the presence of a high GTC on routine breast ultrasonography examination images and mammographic breast composition was investigated. The presence of a high GTC always indicates mammographically dense breasts. A previous study showed that GTC was independently associated with future breast cancer risk in women with dense breasts and reflected lobular involution [8]. However, there are no reports on the association between the presence of a high GTC and mammographic breast composition. To the best of our knowledge, this is the first report showing that a high GTC is a powerful predictor of mammographically dense breasts.

The association between the presence of evident fat lobules in the FGT on routine breast ultrasonography examination images and mammographic breast composition was examined. The presence of evident fat lobules in the FGT always represents mammographic nondense breasts. Moreover, this finding is also a powerful predictor of mammographic nondense breasts.

The current study showed that the simple and robust classification tree with three criteria (presence of a high GTC, presence of evident fat lobules in the FGT, and a cutoff point of 0.93 for the FGT thickness-to-FAT thickness ratio in the groups without a high GTC or evident fat lobules in the FGT) had a high accuracy of 100%. Using the classification tree, breasts can be classified as dense or nondense using routine breast ultrasonography examination rather than relying on sensations and experience.

The current study had several limitations. First, the study population was relatively small. However, this was a preliminary study and the first attempt to develop a classification tree for ultrasonographic breast composition assessment using routine breast ultrasonography examination images. Nevertheless, further validation studies should be performed to determine whether our study results are reproducible on a larger scale. Second, in this study, only a single observer performed ultrasonographic breast composition assessment. This might have introduced bias. Therefore, further studies with multiple readers on this subject must be performed. Third, various ultrasonography machines were used. The effects of using different types of ultrasonography machines on ultrasonographic breast composition assessment was not evaluated. Finally, semiquantitative analysis was performed in this study. However, the recent development of artificial intelligence in imaging processing techniques could improve the interpretation of ultrasonographic breast composition assessment. This can eliminate differences in measurements obtained by a person. Therefore, our results should be considered preliminary.

## Conclusion

The presence of a high GTC can indicate a dense breast. The presence of evident fat lobules in the FGT can represent nondense breasts based on the sonographic breast composition. The cutoff point calculated using Youden’s index was 0.93 for the group without a high GTC or evident fat lobules in the FGT, with a sensitivity of 100% and a specificity of 100%. The simple and robust classification tree comprising three criteria had high accuracy of 100% for classifying nondense or dense breasts on routine breast ultrasonographic examination images.

## Data Availability

The data analysed in this study are available from the corresponding author on resonable request.
